# Endocrine effects of three common gas signaling molecules in humans: A literature review

**DOI:** 10.3389/fendo.2022.1074638

**Published:** 2022-12-09

**Authors:** Wei Qi, Luo Man, Sei Suguro, Yidan Zhao, Heng Quan, Chuoji Huang, Haoran Ma, Haoran Guan, Yizhun Zhu

**Affiliations:** ^1^ School of Pharmacy, Macau University of Science and Technology, Macao, Macao SAR, China; ^2^ State Key Laboratory of Quality Research in Chinese Medicines, Macao, Macao SAR, China; ^3^ The Fifth Affiliated Hospital, Sun Yat-sen University, Zhuhai, China; ^4^ Faculty of Medicine, School of Pharmacy, The Chinese University of Hong Kong, Shatin, China

**Keywords:** gas signaling molecules, endocrine, mechanism of action, pathogenesis, signalling molecules

## Abstract

Gases such as hydrogen sulfide, nitric oxide and sulfur dioxide have important regulatory effects on the endocrine and physiological processes of the body and are collectively referred to as “gas signaling molecules”. These gas signaling molecules are also closely related to Alzheimer’s disease, the inflammatory response and depression. In this paper, we introduce the production and metabolic pathways of NO, H_2_S and SO_2_ in living organisms and review the regulatory functions of gas signaling molecules in the endocrine system and their mechanisms in relation to their clinical applications. This work will provide a basis for finding targets for intervention and establishing novel prevention and treatment strategies for related diseases.

## 1 Introduction

Until the mid-1980s, nitric oxide, hydrogen sulfide and sulfur dioxide were considered to be atmospheric pollutants. The toxicological effects of these gas molecules have been widely demonstrated, and their pharmacological effects and endocrine effects are gradually being revealed. These gas signaling molecules are involved in a wide range of physiological and pathological processes in the neurological, cardiovascular, digestive and metabolic systems of the body. In the organism, L-arginine is metabolized by nitric oxide synthase to produce NO, while sulfur-containing amino acids are metabolized to produce H_2_S and sulfur dioxide. The endogenous production of SO_2_, NO and H_2_S is involved in a wide range of physiological and pathophysiological processes in the body. In mammals, the gas-containing molecules NO, SO_2_ and HS share similar metabolic pathways and have a translational relationship, suggesting that there may be a close link between their regulatory roles in physiological and pathophysiological processes *in vivo*. These connections could provide assistance in the discovery of new signaling pathways or the development of new drugs using these gas signaling molecules. In this paper, we present the production and metabolic pathways of NO, H_2_S and SO_2_ in organisms, with a focus on the regulatory functions of gas signaling molecules in the central nervous system and endocrine system and their mechanisms in relation to their clinical applications, in the context of previous studies and a large data analysis with references.

## 2 Production and metabolism of endogenous gas signaling molecules

In living organisms, endogenous H_2_S and SO_2_ can be produced by the metabolism of sulfur-containing amino acids in response to the action of relevant enzymes. Vascular endothelial cells are able to synthesize NO from L-arginine as an extracellular signal. Endogenous SO_2_ production *in vivo* is mainly based on L-cysteine as a substrate, which is spontaneously broken down into SO_2_ and pyruvate by the action of cysteine dioxygenase and aspartate aminotransferase to produce *β*-sulfinyl pyruvate. In addition, endogenous SO_2_ can be produced directly from endogenous H_2_S by the action of related enzymes. SO_2_ is readily converted to derivatives (HSO_3_^-^/SO_3_^2-^) by water binding in the body and then to sulfate by the action of sulfite oxidase, which is ultimately metabolized by the kidneys and excreted (1).

In mammals, endogenous H_2_S is produced by the enzymatic reactions of cystathione-*β*-synthase (CBS), cystathione-*γ*-lyase (CSE) and 3-mercaptopyruvate transferase (3-MST) using L-cysteine and homocysteine as substrates. Among them, the key enzymes CBS and CSE are 5 ‘phosphopyridoxal-dependent enzymes with a distinct tissue-specific distribution (2–4). CBS is mainly distributed in the central nervous system, while CSE is mainly highly expressed in cardiovascular and other peripheral organs. In addition, 3-MST activity was higher in the brain and erythrocytes. The different tissue distributions suggest that H_2_S may have different physiological roles (5, 6). Endogenous H_2_S exists in the body in two main forms: sodium hydrosulfide (NaHS) and hydrogen sulfide gas (H_2_S). NaHS is ionized and combined with hydrogen ions to convert to H_2_S, a dynamic balance that maintains the pH of the internal environment. Eventually, most of the H_2_S in the body is converted to thiosulfate or sulfate and eliminated from the body (7–9).

## 3 The regulatory role of gas signaling molecules

### 3.1 Regulatory role of NO

#### 3.1.1 Inhibitory effect of NO on inflammation

In recent years, researchers have found that nitric oxide and NO donor molecules have a wide range of applications in the regulation of immunosuppression and anti-inflammation (10–14). Nitric oxide is involved in regulating mitogen-activated protein kinase (MAPK)-related signaling pathways and c-Jun amino-terminal kinase (JNK) signaling pathways, inhibiting the release of inflammatory cytokines such as tumor necrosis factor-*α* and macrophage inflammatory protein factor-1*β* (15–18). In addition, in Jurkat T cells, NO produced by NO-1 has been shown to inhibit T-cell proliferation by downregulating IL-2 secretion (19–21). Several *in vitro* experiments have demonstrated that NO donor molecules, by enhancing endogenous NO activity and NO^-1^ expression, can inhibit LPS-mediated inflammatory responses in RAW264.7 mouse macrophages (22–25). Nitric oxide releasing molecule III(NORM-3) inhibited the inflammatory response of BV-2 microglia induced by thrombin, cytokines and hypoxia. In addition, it has been shown that the anti-inflammatory effect of NORM-3 is significantly enhanced during MAPK pathway inhibition, suggesting that the anti-inflammatory mechanism of NORM-3 is not merely inhibiting enzyme activity but is closely related to deeper cellular signaling pathways. Yu et al. (26) synthesized polymeric nanomicelles formed by the self-assembly of an amphiphilic block copolymer containing an NO donor. The rate and concentration of NO release were dependent on the type of NO donor and the content of sulfhydryl material. He demonstrated the anti-inflammatory effect of NO as a signaling molecule. Incubation of the cells with the NO-releasing micelle significantly reduced the release of SEAP, suggesting that the NO containing nanomicelles could effectively inhibit the LPS-induced inflammatory response.

However, NO donors alone did not exhibit an inhibitory effect but rather exacerbated the inflammatory response (27–30). They speculated that this might be related to the byproducts. Cytotoxicity assays showed that the NO donor was cytotoxic at higher concentrations (IC50 = 650 μmol/L), while the NO-releasing micelle showed no significant cytotoxicity at 0.5 mmol/L. In addition, the NO donor was loaded with phenylboronic acid nanoparticles. In addition, a polymeric nanoparticle containing phenylboronic acid loaded with an NO donor could be prepared as an NO-releasing gas for anti-inflammatory therapy. In a model of LPS-induced inflammation, the NO donor significantly inhibited the secretion of interleukin IL-6 by macrophages. It was also less cytotoxic. NO has a good inhibitory effect on inflammation and has a promising future in the field of inflammation therapy.

#### 3.1.2 Anesthetic effects of NO

NO is an important intracellular and intercellular transmitter and, as an informative substance, is associated with excitatory amino acid-mediated neurotoxicity, consciousness, learning and memory (31, 32). cGMP is then involved in the regulation of various physiological effects. Many studies now suggest that the NO/cGMP signaling system may play an important role in the molecular mechanisms underlying the anesthetic effects of general anesthetics (33).

The majority of intravenous anaesthetics are known to have a close relationship with the NO/cGMP signaling system, and this signaling system plays an important role in the study of the mechanism of general anaesthetic action of these drugs (34, 35). The effects of ketamine, tetracycline and isoproterenol on the NO/cGMP signaling system have been studied more systematically, and it is thought that inhibition of the NO/cGMP signaling system may be an aspect of the mechanism of action of these drugs in general anaesthesia. It has been shown that inhalation anesthetics inhibit the NO/cGMP signaling pathway, reducing the level of consciousness and enhancing anesthesia, analgesia and sedation.

### 3.2 Modulation by H_2_S

The physiological role of endogenous H_2_S in the cardiovascular system has been extensively studied, and its physiological effects on the endocrine system have also received considerable attention due to the tissue-specific nature of its production-dependent enzyme distribution.

#### 3.2.1 H_2_S modulates hippocampal long-duration enhancement

Endogenous H_2_S is thought to be a neuromodulatory substance whose production depends on the wide distribution of CBS and 3-MST in the brain. It was found that physiological concentrations of H_2_S dose-dependently predispose the hippocampal CA1 region to hippocampal long-range memory enhancement (LTP) production. A weak electrical stimulus applied to hippocampal CA1 did not induce LTP, but in the presence of H_2_S, the stimulus facilitated hippocampal LTP production, an effect equivalent to that induced by a tonic electrical stimulus alone. It is worth noting that neither a weak electrical stimulus alone nor a physiological amount of H_2_S alone triggers LTP and that the application of NaSH (the donor of H_2_S) 10 min before and after stimulation also does not lead to LTP production in the hippocampus, so the simultaneous application of H_2_S and stimulation is also an important factor (36).

In addition, the application of the NMDA receptor blocker AP-5 blocked endogenous H_2_S-induced LTP, thus suggesting that the occurrence of endogenous H_2_S-induced LTP is NMDA receptor dependent. Found that the administration of a certain concentration of NaHS (a donor of H_2_S) increased the amount of cyclic adenosine monophosphate (cAMP) in neuronal cells and glial cells *in vitro*, and thus, endogenous H_2_S-induced LTP was dependent on cAMP acting as a second messenger in the relevant cells (37, 38).

#### 3.2.2 Endocrine regulatory effects of H_2_S

The hypothalamic−pituitary−adrenal axis (HPA axis) is an important part of the neuroendocrine system that is involved in the control of stress responses and is closely related to mood disorders and functional disorders (39, 40). In 2000, ex vivo experiments revealed that H_2_S specifically and dose-dependently inhibited the release of adrenocorticotropin-releasing hormone (CRH) in the hypothalamus and that H_2_S significantly inhibited KCl-induced CRH release, which may be related to the regulation of K^+^ channels (activation) and Ca^2+^ channels (inhibition) by H_2_S. High concentrations of H_2_S exhibited its toxic gas effect, a process that was associated with the inactivation of cytochrome oxidase and cytochrome activity by H_2_S. The hypothalamic paraventricular nucleus (PVN) is known for its activation of the HPA axis through CRH secretion, and CBS enzymes are expressed in the PVN. Zhang et al. (41) identified a new brain fat feedback axis, the leptin-FOXO3a-hypothalamic CBS/H_2_S system, which regulates energy homeostasis by modulating neuroendocrine hormone (TRH, ACTH) and leptin levels. The proposal of this axis complements the important role of H_2_S in the neuroendocrine system.

In addition to this, hydrogen sulphide can also regulate endocrine function by regulating glucose and lipid metabolism in adipocytes Adipocyte uptake of excess energy and its storage as fat is a fundamental function of adipocytes. In human mature adipocytes, hydrogen sulphide donors dose-dependently inhibit adipocyte basal and insulin-stimulated glucose uptake, and conversely, CSE inhibitors promote adipocyte glucose uptake. The application of PI3K inhibitors blocked this effect, suggesting that the function of hydrogen sulfide in regulating adipocyte glucose uptake is partially mediated by post-insulin receptor signaling. TNF*α* upregulated CSE-hydrogen sulphide upregulation in 3T3L1 adipocytes and induced a downregulation of adipocyte glucose uptake and a decrease in insulin responsiveness, but hydrogen sulphide donors reversed TNF*α*-induced insulin resistance. Hydrogen sulfide donors or precursors can inhibit homologous phosphatase-tensin (PTEN) phosphorylation of PI3K-Akt-PKC*ζ*/*λ* signaling and promote glucose transporter 4 expression to increase adipocyte glucose utilization; they can also increase insulin receptor phosphorylation and activate Akt signaling to promote sugar uptake. In addition, vitamin D promoted increased sugar uptake in insulin-resistant adipocytes by inducing the expression of CSE-hydrogen sulfide. Activation of peroxisome proliferator-activated receptor*γ*(PPAR*γ*) promotes glucose transporter translocation to the cell membrane, facilitating glucose uptake, and also promotes insulin signaling and transcription of multiple genes for fatty acid synthesis, improving insulin resistance. Hydrogen sulfide modifies the sulfhydration of cysteine residues at position 139 of PPAR*γ*, increasing its transcriptional activity and also promoting glucose uptake in adipocytes. Thus, endogenous hydrogen sulfide can stabilize adipocyte glucose uptake, and when CSE-hydrogen sulfide is sufficient under physiological conditions, additional hydrogen sulfide can inhibit glucose uptake and utilization; while when stimuli such as high glucose suppress the endogenous hydrogen sulfide system and cause insulin resistance, hydrogen sulfide supplementation can improve their glucose metabolism and restore their regulation of energy. Adipocytes convert excess glucose into fatty acids which are then esterified into triglycerides and stored in lipid droplets. During starvation or stress, triglycerides are hydrolysed and free fatty acids are released, while free fatty acids are *β*-oxidised and ATP is released to supply the body’s needs. Inhibitors of CSE can activate PKA, which leads to the activation and translocation of hormone-sensitive lipase to the lipid droplet surface, and phosphorylation of the lipid droplet envelope protein perilipin-1, which exposes the lipid droplet to the hormone-sensitive lipase binding site and accelerates lipolysis. Conversely, hydrogen sulphide donors inhibit lipolysis.

The addition of hydrogen sulphide donors (NaHS and GYY4137) during the differentiation of adipocytes into adipocytes can increase the expression of differentiation markers such as AP2 and CEBP and increase the number of adipocytes; the application of CBS inhibitor AOAA or CSE inhibitor PPG can partially block their differentiation, and the blocking effect of PPG is more significant. Overexpression of CSE increased the differentiation of adipocytes, while knockdown of endogenous CSE significantly inhibited their differentiation. On the one hand, hydrogen sulphide acts as a phosphodiesterase inhibitor, increasing intracellular cAMP levels and activating CEBP; on the other hand, hydrogen sulphide directly thiolates the cysteine residue at 139 of PPAR*γ*, increasing its nuclear translocation and DNA binding activity and promoting the expression of lipogenic genes. Sulfhydrylation of the 139 cysteine residue of PPAR*γ* may regulate its protein stability and increase the expression level of PPAR*γ* protein. It is suggested that endogenous hydrogen sulfide may act as an energy switch to regulate glycolipid conversion in adipocytes. Under physiological conditions, excess hydrogen sulfide inhibits glucose uptake and promotes the conversion of sugar into lipids for storage; when pathological conditions such as hyperglycemia and hyperlipidemia occur, CSE-hydrogen sulfide is reduced, resulting in inactivation of PPAR*γ*, which inhibits insulin signaling and causes a decrease in glucose uptake, while inducing lipolytic reactions, releasing excess free fatty acids, causing ectopic deposition of fat, and promoting oxidative stress and inflammation in tissue cells, It also contributes to oxidative stress and inflammation in tissue cells, exacerbating disease damage.

Hydrogen sulphide can also cause adipocytes to secrete a variety of adipokines and inflammatory factors. In animals in which adipocytes were induced with hydrogen sulphide, plasma leptin levels were increased. In differentiated adipocytes from rats, overexpression of CSE increased leptin expression. Under high glucose induction, overexpression of CSE increased adiponectin expression in 3T3L1 differentiated adipocytes and decreased MCP-1 expression, but had no effect on TNF*α*. Therefore, hydrogen sulfide, as an autocrine factor, can promote the expression of protective adipokines, inhibit the release of inflammatory factors and regulate endocrine function.

### 3.3 The regulatory role of SO_2_

With the discovery of the endogenous SO_2_ production system, the dual regulatory functions of SO_2_ in the central nervous system are gradually being revealed.

#### 3.3.1 SO_2_ and oxidative stress injury

It was found that SO_2_ decreased the activity of antioxidant enzymes and increased the content of markers reflecting oxidative damage in multiple organs of the brain, lung and heart of mice. Fu et al. (42) found that in a mouse model of pulmonary hypertension and myocardial ischaemia−reperfusion injury, SO_2_ increased the levels of antioxidants and decreased oxidants (ROS, H_2_O_2_), and this effect was dependent on SO_2_ upregulating the myocardial H_2_S/CSE pathway and downregulating the NO/iNOS pathway. In a colitis model in which trinitrobenzenesulfonic acid induced a disturbance in the redox status of colonic tissue, Ran et al. (43) discovered that SO_2_ (Na_2_SO_3_:NaHSO_3_, 3:1) treatment restored the expression levels of antioxidant molecules while reducing the expression of GST and GSH. SO_2_ reduced CCH injury in a model of chronic cerebral ischemia (CCH) by increasing the antioxidant capacity of the hippocampus. All of these studies indicate that SO_2_ may reverse the oxidative damage process in certain diseases due to its antioxidant properties.

#### 3.3.2 SO_2_ and the inflammatory response

In earlier years, the reduction of sulfite from SO_2_ was widely demonstrated as an inflammatory mediator in models of acute lung injury. Schalley et al. (44) found that SO_2_ significantly reduced the inflammatory response to lipopolysaccharide-induced ALI by downregulating the expression of Raf-1, MEK-1 and p-ERK in the MAPK signaling pathway and inhibiting IL-1*β* and IL-6 production during ALI. Schalley found that in colitis, SO_2_ reduced inflammation by decreasing TNF-*α* expression and NF-*κ*B activation. Macrophage activation-mediated inflammatory responses are an important component of inflammatory diseases. The discovery that the endogenous SO_2_/AAT pathway exists in macrophages and that macrophage-derived SO_2_ inhibits macrophage-mediated inflammatory responses *via* the NF-κB pathway opens new horizons for the study of anti-inflammatory therapy and mechanisms.

#### 3.3.3 SO_2_ and apoptosis

Lin et al. (45) found that downregulation of the endogenous SO_2_/AAT system is an important initiator of hypoxia-stimulated apoptosis in human pulmonary artery endothelial cells, which is mediated by upregulation of bcl-2. This suggests that the endogenous SO_2_/AAT system may regulate the apoptosis of neurons in epileptic rats through the activation of the endoplasmic reticulum stress-related signaling pathway by PERK and affect epileptogenesis (46). Recently, the anti-apoptotic effect of SO_2_ was demonstrated, and SO_2_ significantly reduced the rate of cell necrosis and apoptosis in the hippocampal CA1 region of transient whole brain ischemia/reperfusion (I/R) rats and improved the learning memory impairment induced by I/R (47, 48). The above studies suggest that the regulatory effect of SO_2_ on apoptosis may be dual in nature.

## 4 Mechanisms of regulation of disease by gas signaling molecules

### 4.1 Gas signaling molecules and Parkinson’s disease

The pathogenesis of Parkinson’s disease (PD) involves degeneration of the substantia nigra-striatal dopamine pathway, significantly reduced dopamine levels and dysfunction of the Ach system. Chatterjee (49) found that endogenous H_2_S levels were significantly reduced in the substantia nigra (SN) in a 6-hydroxydopamine (6-OHDA)- and rotenone-induced PD rat model. NaHS reverses neurodegeneration by inhibiting NaHS and reverses neurotoxin-induced neurodegeneration by inhibiting NADPH oxidase and anti-microglia activation. In addition, H_2_S reversed 6-OHDA neurological damage through a nigrostriatal leptin-dependent pathway, enhancing the Warburg effect (50).

### 4.2 Gas signaling molecules and Alzheimer’s disease

Alzheimer’s disease (AD) is a neurodegenerative disorder characterised by neuronal fibrillary tangles, with the typical clinical manifestation being a reduction in cognitive ability in the brain. The main cause of Alzheimer’s disease is the deterioration of cognitive and memory functions due to central neurodegeneration and characteristic brain degeneration. Homocysteine (Hcy) is a high risk factor for Alzheimer’s disease and high expression of Hcy is associated with characteristic brain degeneration, which in turn causes Alzheimer’s disease in patients. Wu, Y (51) found that serum levels of Hcy were significantly higher in AD patients than in normal subjects. It was found that the level of S-adenosylmethionine (AdoMet) in the brains of patients was 0.18 ± 0.04 nmol/mg, which was much lower than the normal level of 0.52 ± 0.07 nmol/mg. This was due to the low level of AdoMet in the brains of AD patients, which affected CBS activity and thus reduced the synthesis of H_2_S. In a severe AD model, H_2_S supply reduced A*β* deposition, hyperphosphorylation of p-APP (the precursor protein of A*β*) and Tau isoforms, and the inflammatory response to improve cognitive activity (52). This provides a new idea for the mechanism of the protective effect of H_2_S on AD.

### 4.3 Gas signaling molecules and cancer

Earlier, researchers identified a pro-apoptotic effect of NO gas on tumour cells. p53 inhibits apoptosis by causing mutations in p53. p53 regulates redox stress, induces cell death, inhibits cell migration and angiogenesis, and limits stem cell renewal, thereby inhibiting tumorigenesis (53). p53 is regulated by ubiquitin ligase (Mdm2), which targets Mdm2 targets p53 for degradation and directly inhibits p53 activity by binding to the transcriptional activation domain. The mutation rate of the tumour suppressor gene p53 in human malignant tumours is 30%-50%, and the presence of one mutated p53 protein in different combinations of p53 tetramers effectively interferes with the function of the entire tetramer, rendering its apoptotic function completely inactive (54). Thus, mutated p53 not only eliminates wild-type p53 function but also significantly impairs wild-type p53 function.

**Figure 1:**
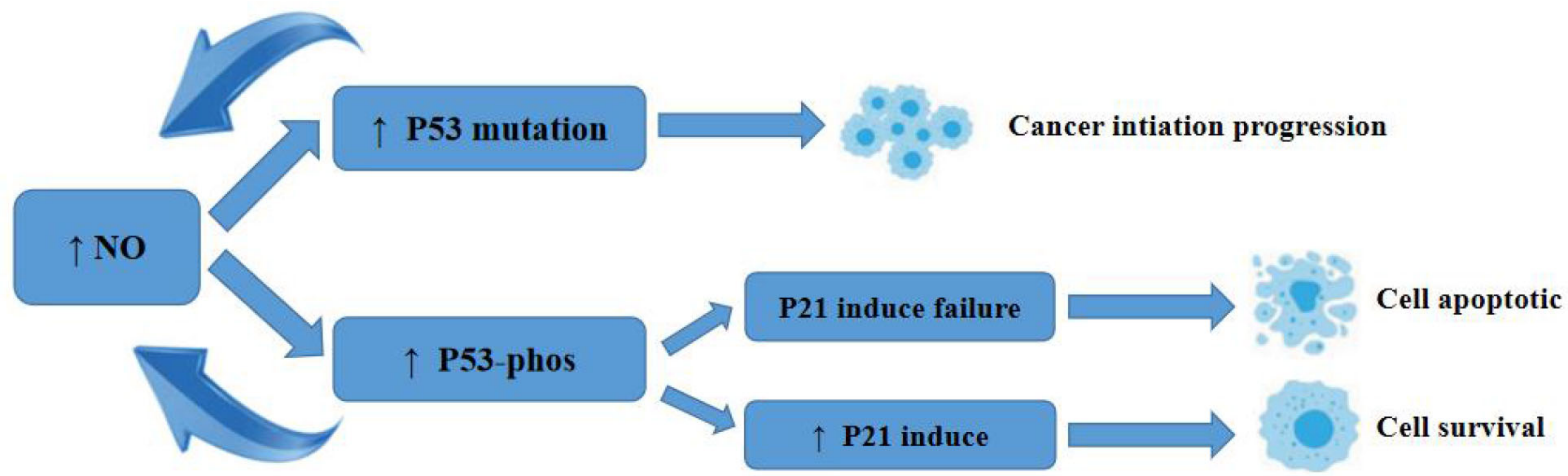
Schematic diagram of the mechanism of action of NO on P53.

NO causes p53 mutation at concentrations below 400 nmol/L, and when NO contributes to p53 mutation, the mutated p53 has no tumour suppressive activity and impairs wild-type p53 function, leading to cell survival (55). Chromogranin C and ultimately activation of caspase-3 can also effectively inhibit apoptosis. The process of this role is shown in **Figure 1**.

### 4.4 Gas signaling molecules and depression

Zhao et al. (56) used a rat model of chronic unpredictable stress (CUMS) depression and found that CUMS induced a downregulation of hippocampal CBS expression and a decrease in H_2_S production in rats. Exogenous H_2_S treatment significantly improved CUMS-induced depressive-like behaviour. Regarding the protective mechanism of H_2_S, Begara et al. (57) showed that the BDNF-TrkB pathway mediated the antidepressant effects of H_2_S in CUMS-induced rats by inhibiting hippocampal endoplasmic reticulum stress. H_2_S enhanced antioxidant capacity and exerted antidepressant and anxiolytic effects in streptozotocin-treated diabetic rats.

### 4.5 Gas signaling molecules and epilepsy

Fix et al. (58) investigated whether the effect of SO_2_ on brain injury in rats with recurrent febrile convulsions (FS) was dose dependent. This study found that the SO_2_/AAT system is involved in the FS process and that in a rat FS model, low concentrations of SO_2_ (Na_2_SO_3_:NaHSO_3_, 3:1, 1-10 μmol/kg) pretreatment reduced neuronal injury and apoptosis, whereas AAT inhibitors or high concentrations of SO_2_ (Na_2_SO_3_:NaHSO_3_, 3:1, 100 μmol/kg) produced neuronal damage effects. However, Lv et al. (59) found that in epileptic rats, endogenous SO_2_ induced apoptosis in hippocampal neurons *via* the PERK signaling pathway. Inhibition of endogenous SO_2_ production attenuated neuronal apoptosis at the beginning of seizures, but this attenuating effect only delayed the onset of neuronal apoptosis but did not prevent neuronal apoptosis.

## 5 Discussion

As endogenous gas signaling molecules, the regulatory effects of endogenous NO, H_2_S and SO_2_ on many systems in the body and their mechanisms of action on various diseases have been elucidated, but their regulatory functions and mechanisms in the endocrine system have not been fully understood (60). Recent studies at home and abroad have shown that NO, H_2_S and SO_2_ act by similar but slightly different mechanisms and that the mechanisms of action differ in different systems.

### 5.1 Mechanism of action of NO in the endocrine system

In cells, NO is produced by nitric oxide synthase in three forms (neuronal NO synthase, nNOS or NOS I); inducible NO synthase, i NOS or NOS II and endothelial NO synthase, eNOS or NOS III (61). These enzymes use arginine as a substrate to produce NO and citrulline. The three NOS have similar molecular structures: an N-terminal oxygenase region, a calmodulin-binding region and a C-terminal reductase region. e NOS and nNOS are both activated by a rise in intracellular calcium. eNOS is highly expressed in vascular endothelial cells, while n NOS is mainly found in the nervous system, where the synthesis and release of NO from neurons is defined as a function of the presence of a coenzyme. The neurons that synthesize and release NO are defined as nitroergic neurons. In the mammalian autonomic nervous system, nNOS is usually found in parasympathetic postganglionic neurons. iNOS was first identified in macrophages and is induced in macrophages and other cells and tissues by bacterial lipopolysaccharides and growth inhibitors. iNOS is metabolized in the body mainly to NO_2_^-^ and NO_3_^-^, with a small amount excreted by respiration.

In the endocrine system, NOS, as the main synthesizing enzyme of NO, plays an important role in maintaining the homeostasis of the endocrine system. These results suggest that NO can inhibit the development of atherosclerosis and other diseases.

The affinity of NO for iron in proteins can inhibit the catalytic activity of many key iron-containing enzymes, including iron-sulfur cluster-dependent enzymes that catalyse the mitochondrial respiratory chain, nucleotide reductase, and cis-aconitase. In addition, NO generated by NOS can directly interfere with the synthesis of DNA in target cells, leading to DNA strand breaks and fragmentation. In parasitic microorganisms and specific tumour cells, these effects may contribute to the cytostatic and toxic effects of NO.

NO activates GC in vascular smooth muscle cells to increase the cGMP concentration and decrease the intracellular free Ca^2+^ concentration, resulting in vasodilation. In addition, NO inhibits cell proliferation and counteracts vasoconstrictive substances. NO regulates a large number of mitochondrial haemoglobin proteins by a similar mechanism. The s-nitrosylation modification is an important way in which NO exerts its biological function. This is a posttranslational modification similar to phosphorylation and acetylation, in which NO or peroxynitrite acts on the cysteine sulfhydryl group (-SH) of a protein to produce -SNO. In addition, S-nitrosylated glyceraldehyde-3-phosphate dehydrogenase activates downstream apoptotic signals and regulates cell and tumour growth.

Therefore, future research should focus on the use of NO’s affinity for proteins to inhibit tumour cell growth in tumour cells. At the same time, the focus should also be on the inhibitory effect of NO on the development of diseases such as atherosclerosis. The regulatory effects of NO on the endocrine system and some cells can be used to inhibit tumour growth or inhibit disease progression.

### 5.2 Mechanism of action of H_2_S in the endocrine system

H_2_S in humans is produced by the enzymatic reactions of cystathionine and homocysteine as substrates, cystathionine beta synthase (CBS), cystathionine gamma lyase (CSE), and 3-mercaptopyruvate transsulfurase (3MST). The distribution of these enzymes is tissue specific, with CBS highly expressed in the nervous system, CSE mainly in cardiovascular tissue, and both in the liver and kidney. In cellular mitochondria, H_2_S is rapidly oxidized to thiosulfate (S_2_O_3_^2-^) and subsequently converted to sulfite (SO_3_^2-^) and the more stable sulfate (SO_4_^2-^).

Recent studies have shown that H_2_S signals through S-mercapturised proteins, a process in which H_2_S acts on cysteine sulfhydryl groups (-SH) of proteins to produce -SSH, which can be detected by a modified biotin-switch method. Under physiological conditions, many proteins in mammalian liver are modified by S-mercapturisation, and approximately 10%-25% of endogenous GADPH, *β*-tubulin and actin in liver are mercapturised, five to ten times more than by nitroxylation. parkin proteins have cysteine sites that can be S-mercapturised, and under physiological conditions, they can be modified by exogenous hydrogen sulfide. This suggests that reduced parkin S-mercapturization may be involved in the development of PD (62). In contrast, the free thiol group at the cysteine 38 site of NF-*κ*B p65 can be modified by S-mercapturization, which may be a key mechanism for H_2_S inhibition of oxidized LDL-induced inflammation in macrophages. Further studies may reveal more S-mercapto-modified proteins and their corresponding functional alterations.

Although considerable progress has been made in the study of the mechanism of action of H_2_S, the molecular targets of its action are still not well understood. Earlier studies have supported the possibility that the ATP-sensitive potassium channel (KATP) may be one of the functional targets of H_2_S. However, in mice with knockout of the KATP channel subunit Kir6.2, H_2_S was still able to protect against MPTP-induced dopamine neuron deficiency, suggesting that H_2_S does not act directly on the KATP channel; therefore, further studies are needed to investigate the mechanism of action of hydrogen sulfide.

Although the molecular targets of H_2_S have not yet been identified, current studies have found that H_2_S has an inhibitory effect on dopamine neuron deficits as well as a modulatory effect on validation, and further research should focus on investigating the mechanisms underlying the inhibitory effect of H_2_S on dopamine neuron deficits, with a view to applying these mechanisms in clinical treatment. At the same time, the mechanism of H_2_S modulation of inflammation should be further investigated to develop H_2_S-based inflammation-modulating drugs.

### 5.3 Mechanism of action of SO_2_ in the endocrine system

Mammals produce SO_2_ through the metabolism of sulfur-containing amino acids or through the oxidation of hydrogen sulfide (H_2_S), catalyzed by aspartate aminotransferase (AAT). Endogenous SO_2_ is synthesized in both vascular endothelial and smooth muscle cells; mainly in vascular endothelial cells, endogenous SO_2_ synthesized *in vivo* can be converted to endogenous sulfite and bisulfite to produce biological effects (63).

Endogenously produced SO_2_ and SO_2_ derivatives have important cardiovascular physiological effects in the regulation of vascular tone and cardiac function, and SO_2_ is also pathophysiologically important in many cardiovascular diseases, such as hypertension, atherosclerosis, pulmonary hypertension, ischaemia−reperfusion injury and myocardial injury. However, the biological mechanisms by which endogenous SO_2_ regulates different cardiovascular diseases and other cardiovascular effects need further investigation. Studies on the cardioprotective function of SO_2_ provide new therapeutic strategies based on the regulation of H_2_S and SO_2_ production. However, the functional and signaling pathways associated with CSE/CBS/MPST and AAT in the cardiovascular system need to be investigated in more depth. In addition, the design of physiologically concentrated SO_2_-controlled drugs is urgently needed, as stable and reliable SO_2_ donors are not only useful research tools but also potential drugs for the treatment of endocrine diseases. Future research will focus on the development of SO2-based drugs for the treatment of endocrine disorders through the effects of SO2 and its derivatives on cardiovascular diseases and the mechanisms that regulate endocrine. To date, many cardiovascular studies with SO_2_ have been conducted in rats and mice, and clinical evidence is lacking. Studying the effects of H_2_S and SO_2_ in large animal models with cardiovascular disease characteristics similar to those of humans would be more useful for translation to clinical trials.

## 6 Conclusions

Through a keyword search in Web Of Science, we collected, screened, integrated and summarised previous studies to clarify the regulatory functions and interaction mechanisms of gas signaling molecules in the endocrine system, and analysed their mechanisms of action in various disease processes, providing a basis for identifying targets for intervention and therapeutic strategies for related diseases.

## Author contributions

Methodology: WQ, LM, SS, YdZ, HQ, CH, HM, HG, and YzZ. Validation: WQ, LM, SS, YdZ, HQ, CH, HM, HG, and YzZ. Formal analysis: WQ, LM, SS, YdZ, HQ, CH, HM, HG, and YzZ. Investigation: WQ, LM, SS, YdZ, HQ, CH, HM, HG, and YzZ. Resources: WQ, LM, SS, YdZ, HQ, CH, HM, HG, and YzZ. Data curation: WQ and LM. Writing - original draft: WQ. Writing - review & editing: WQ and LM. Supervision: WQ and LM. Project administration: WQ. All authors contributed to the article and approved the submitted version.
